# Association between *APOE* Genotype and Change in Physical Function in a Population-Based Swedish Cohort of Older Individuals Followed Over Four Years

**DOI:** 10.3389/fnagi.2016.00225

**Published:** 2016-10-04

**Authors:** Ingmar Skoog, Helena Hörder, Kerstin Frändin, Lena Johansson, Svante Östling, Kaj Blennow, Henrik Zetterberg, Anna Zettergren

**Affiliations:** ^1^Neuropsychiatric Epidemiology Unit, Department of Psychiatry and Neurochemistry, Institute of Neuroscience and Physiology, Sahlgrenska Academy, University of GothenburgMölndal, Sweden; ^2^Clinical Neurochemistry Lab, Department of Psychiatry and Neurochemistry, Institute of Neuroscience and Physiology, Sahlgrenska Academy, at the University of GothenburgMölndal, Sweden; ^3^Department of Molecular Neuroscience, Institute of Neurology, University College of LondonLondon, UK

**Keywords:** physical function, grip strength, gait speed, *APOE* 𝜀4 allele, dementia

## Abstract

The association between decline in physical function and age-related conditions, such as reduced cognitive performance and vascular disease, may be explained by genetic influence on shared biological pathways of importance for aging. The apolipoprotein E (*APOE*) gene is well-known for its association with Alzheimer’s disease, but has also been related to other disorders of importance for aging. The aim of this study was to investigate possible associations between *APOE* allele status and physical function in a population-based longitudinal study of older individuals. In 2005, at the age of 75, 622 individuals underwent neuropsychiatric and physical examinations, including tests of physical function, and *APOE*-genotyping. Follow-up examinations were performed at age 79. A significantly larger decline in grip strength (*p* = 0.015) between age 75 and 79 was found when comparing *APOE* 𝜀4 allele carriers with non-carriers [10.3 (±10.8) kg versus 7.8 (±10.1) kg]. No association was seen with decline in gait speed, chair-stand, or balance. The association with grip strength remained after correction for cognitive and educational level, depression, cardiovascular disease, stroke, and BMI.

## Introduction

The apolipoprotein (*APOE)* gene, encoding APOE involved in lipid metabolism, is a well-established risk factor for Alzheimer’s disease (AD) (Corder et al., 1993; Poirier et al., 1993; Kandimalla et al., 2011, 2013; Yu et al., 2014). The gene has three common alleles (𝜀2, 𝜀3, and 𝜀4), and carriers of the 𝜀2 allele are at lower risk, while 𝜀4 allele carriers are at higher risk, of the disorder (Bertram et al., 2007). In cognitively healthy individuals, associations between *APOE* 𝜀4 and worse performance on cognitive tests, especially in old populations, have been reported (Caselli et al., 2009; Wisdom et al., 2011; Davies et al., 2014). Further, the 𝜀4 allele is a risk factor for other conditions that mainly affect older individuals, such as atherosclerosis (Zhu et al., 2016) and cardiovascular and cerebrovascular disease (Lehtinen et al., 1995; McCarron et al., 1999; Zlokovic, 2013). Moreover, in a recent study, our research group found that *APOE* 𝜀4 predicts future depression in older persons (Skoog et al., 2015).

Physical function, objectively assessed by tests of grip strength, gait speed, chair-stand, and standing balance, has been shown to be a good predictor of several conditions, such as cognitive performance (Deary et al., 2006; Boyle et al., 2009), cardiovascular disease (Leong et al., 2015), activities of daily living (ADL) and disability, as well as mortality (Abellan van Kan et al., 2009; Cooper et al., 2010). The association between decline in physical function and age-related conditions may be explained by genetic influence on shared biological pathways of importance for aging. In view of the above mentioned associations between the *APOE* gene and several age-related disorders, it is not farfetched to suggest that this gene also affects physical function.

Few studies on the relation between the *APOE* gene and physical function in old age have been performed (Melzer et al., 2005; Buchman et al., 2009; Batterham et al., 2013; Vasunilashorn et al., 2013; Verghese et al., 2013; Alfred et al., 2014), and the results are inconsistent. The purpose of the present study was to investigate possible associations between *APOE* allele status and measures of physical function in a Swedish population-based longitudinal study of older individuals.

## Materials and Methods

### Study Sample

Participants originate from two epidemiological studies in Gothenburg, Sweden, the Prospective Population Study of Women (PPSW) and the Gerontological and Geriatric Population Studies (H70), which were merged in 2000–2001 to become one study. The samples have been described in detail previously (Steen and Djurfeldt, 1993; Bengtsson et al., 1997; Skoog, 2004; Karlsson et al., 2009). The participants were sampled from the Swedish Population Register on the basis of birth date and were born in 1930. Adults living in private households and in residential care were included. Examinations were done at an outpatient department or in the participants’ home. In 2005, there were 1287 eligible individuals and 827 agreed to participate (response rate 64%). Six hundred and thirty eight individuals participated in at least one test of physical function, and 622 (97%) of those gave informed consent to participate in genetic analyses. In 2005, tests of physical function were only done at the outpatient department. Thus, no individuals were included among those examined through home visits (*n* = 129). In 2009, there were 1108 eligible individuals and 662 agreed to participate (response rate 60%). Six hundred and ten participated in the fitness tests and 602 (99%) of them consented to genetic analyses. In 2009, no tests of gait speed were done among individuals examined at home visits (*n* = 197). Among individuals included in the genetic analyses, 448 participated in at least one test of physical function in both 2005 and 2009. The study was approved by the Regional Ethical Review Board in Gothenburg (approval numbers: S 069-01, T 453-04, 075-09), and written informed consent was obtained from all participants and/or their relatives in cases of dementia.

### General Examinations and Diagnoses

Clinical examinations included comprehensive social, functional, physical, neuropsychiatric, and neuropsychological examinations, as well as close informant interviews (Skoog, 2004). All examinations were carried out by health professionals, such as nurses or physiotherapists. Dementia was diagnosed by geriatric psychiatrists according to the Diagnostic and Statistical Manual of Mental Disorders 3rd Edition Revised (DSM-III-R; APA, 1987), based on symptoms rated during the neuropsychiatric examinations and information from the close informant interviews, as described previously (Skoog et al., 1993, 2015; Guo et al., 2007). Cognitive function was assessed with the Mini-Mental State Examination (MMSE) (Folstein et al., 1975). Major and minor depression were diagnosed based on the neuropsychiatric examination according to the Diagnostic and Statistical Manual of Mental Disorders 4th Edition (DSM-IV) (APA, 1994) criteria for minor depression, and Diagnostic and Statistical Manual of Mental Disorders 5th edition (DSM-5) (APA, 2013) for major depression. Cardiovascular disease was defined as angina pectoris according to the Rose criteria (Rose, 1962) and/or myocardial infarction (MI) according to self-reported history or ECG criteria (Minnesota code 1-1-1 to 1-2-5 or 1-2-7). The diagnosis of stroke/TIA was based on information from self-reports, close informants and the Swedish hospital discharge register, as described previously (Liebetrau et al., 2003). Diabetes was diagnosed based on self-reported history and use of antidiabetic drugs. Cholesterol was measured with standard methods at the laboratory at Sahlgrenska University Hospital. Regarding physical activity, participants were asked about level of physical activity in their leisure time based on the Saltin–Grimby Physical Activity Level Scale (Grimby et al., 2015). The scale is a combined frequency–intensity measure including the following options: (1) ‘Almost totally inactive’ (e.g., reading, watching TV, going to the movies), (2) ‘Some physical activity at a minimum of 4 h/week’ (e.g., bicycling, walking to/from workplace, or during leisure time, walking with family), (3) ‘Regular physical activity’ (e.g., gardening, golfing, running, keep-fit exercise, tennis, dancing), and (4) ‘Regular intense physical activity and contests’ (e.g., running several times/week, swimming several times/week, competitive sports).

### Tests of Physical Function

Grip strength was tested with a Jamar dynamometer at an elbow angle of 90 degrees and with the shoulder joint in a neutral position. The test was repeated three times for each hand, and the highest value of the best hand was used as outcome (kg). The method has been shown to have high intra- and inter-test reliability (Peolsson et al., 2001) and validity (Bellace et al., 2000). Self-selected and maximum gait speed for 20-m indoors with standing start (meter/second) were measured (Frändin and Grimby, 1994). The walking test has shown good intra- and inter-rater reliability (Connelly et al., 1996). Timed chair-stand measures mobility by testing the ability to stand up and sit down from a chair five times in a row as quickly as possible. The total time (seconds) was used as outcome. The test displays discriminative and concurrent validity properties (Whitney et al., 2005). Balance was tested by measuring the ability to stand on one leg without shoes, for a maximum of 30 s (Stones and Kozma, 1987). The test was interrupted if the individual moved from the standardized position. Three trials for each leg were allowed, and the best result from the best leg was used for analysis.

### Genotyping

Blood samples were collected and the SNPs rs7412 and rs429358 in *APOE* (gene map locus 19q13.2) were genotyped with KASPar^®^ PCR SNP genotyping system (LGC Genomics, Hoddesdon, Herts, UK) or by mini-sequencing (as previously described in detail; Blennow et al., 2000). Genotype-data for these two SNPs were used to unambiguously define 𝜀2, 𝜀3, and 𝜀4 alleles. The genotyping success rate was >95% (genotyping failed for 13 individuals in 2005 and 21 individuals in 2009).

### Statistical Analysis

Differences in distribution or mean value of sample characteristics between age 75 and 79 were investigated with Fischer’s exact test or *t*-test, respectively. The relation between change in physical function, between age 75 and 79, and *APOE* 𝜀4 status, as well as cross-sectional associations between physical function and *APOE* 𝜀4 status, were analyzed with linear regressions including sex as a covariate. The association with change in grip strength was further analyzed using body mass index (BMI) at baseline (age 75), diabetes at any occasion (i.e., at age 75 or 79), total cholesterol at baseline, cardiovascular disease at any occasion, stroke up to age 79, depression (major and minor) at any occasion, MMSE score at baseline, change in MMSE score, and educational level [dichotomized as compulsory (7 years), or more] as covariates. The cross-sectional association with grip strength found at age 79 was further analyzed using BMI at age 79, diabetes at age 79, total cholesterol at age 79, cardiovascular disease at age 79, stroke up to age 79, depression at age 79, MMSE score at age 79, and educational level as covariates. As a first step, both in the longitudinal and cross-sectional analysis, covariates were included in four different models; model one included covariates related to cognition (i.e., MMSE score and educational level), model two included covariates related to mental health (i.e., major and minor depression), model three included cardiovascular disease and stroke, and model four included BMI, diabetes and total cholesterol. As a final step, all covariates were included in the same model. Further, all analyses were re-done after excluding individuals with a dementia diagnosis up to year 2009. In addition, we performed the analyses only including individuals who reported that they were physically active (option 2–4 on the Saltin–Grimby Physical Activity Level Scale), and to be included in the longitudinal analyses an individual should have been physically active at both examinations. We also did sensitivity analyses, where only those who were examined at the out-patient department were included, as gait speed was not done at any home visits. All analyses were performed using SPSS version 22 for Windows. The power of the study varied between 90 and 58%, depending on the outcome (largest power for detecting an association with maximum gait speed and lowest power for detecting an association with chair-stand). The power-calculations were based on the assumption that the difference in decrease between *APOE* 𝜀4 carriers and non-carriers would be 3 kg (*SD*: 10 kg) for grip strength, 0.05 m/s (*SD*: 0.15 m/s) for regular gait speed, 0.1 m/s (*SD*: 0.25 m/s) for maximum gait speed, 1 s (*SD*: 4 s) for chair-stand, and 3 s (*SD*: 10 s) for balance. The power-calculations were done using SAS version 9.4.

## Results

Relevant characteristics of the samples at age 75 and/or 79 are summarized in **Table 1**. The difference in percentage of *APOE* 𝜀4 carriers between age 75 and 79 (**Table 1**) can at least partly be explained by the fact that a non-significantly larger percentage of *APOE* 𝜀4 carriers, who participated at age 75, had died or declined to participate at age 79, compared to non-carriers. A significant association (*p* = 0.015) was found between possession of the *APOE* 𝜀4 allele and larger decline in grip strength between age 75 and 79 (**Table 2**). No association was found between *APOE* 𝜀4 status and change in gait speed, chair-stand, or balance. In the cross-sectional analyses, no association was found between *APOE* 𝜀4 status and physical function at age 75, while a significant association was found with grip strength (*p* = 0.006) at age 79 years (**Table 3**). Analyses of the interaction between *APOE* 𝜀4 and age showed that the effect of the 𝜀4 allele on grip strength was significantly larger at age 79 than at age 75 (*p* = 0.033). Both the longitudinal and the cross-sectional association with grip strength remained after correction for MMSE score, educational level, depression, cardiovascular disease, stroke, BMI, diabetes, and total cholesterol. Further, exclusion of all cases with dementia, or inclusion of only physically active individuals, did not change the results. Also, the main results did not change when only individuals who were examined at the out-patient department were included. Moreover, after excluding individuals with the 𝜀2𝜀4 genotype (*n* = 6 in the longitudinal analysis and *n* = 9 in the cross-sectional analysis at age 79), the relation between grip strength and *APOE* 𝜀4 became stronger [mean 19.2 (±10.5) kg for 𝜀4 carriers and 23.0 (±11.9) kg for 𝜀4 non-carriers among 79 year olds (*p* = 0.001), and a decline of 11.4 (±9.7) kg for 𝜀4 carriers and 7.8 (±10.1) kg for 𝜀4 non-carriers (*p* = 0.001) between 75 and 79 years of age].

**Table 1:** Characteristics of the study sample.

	75 year olds (*n* with genotype = 609)	79 year olds (*n* with genotype = 581)	*p*-value
Women, *n* (%)	364 (59.8)	347 (59.7)	1.00
*APOE* aaa4 carriers, *n* (%)	167 (27.4)	118 (20.3)	0.004
Body mass index, mean (*SD*)	26.8 (4.2)	26.1 (4.2)	0.007
Cardiovascular disease*, *n* (%)	95 (15.6)	97 (16.7)	0.64
Diabetes, *n* (%)	78 (12.8)	80 (13.8)	0.67
Cholesterol (mmol/l), mean (*SD*)	5.33 (1.07)	5.39 (1.09)	0.34
Stroke, *n* (%)	52 (8.5)	66 (11.4)	0.12
Dementia, *n* (%)	16 (2.6)	37 (6.4)	0.002
MMSE, mean (*SD*)	27.6 (1.8)	27.7 (3.4)	0.52
Education more than compulsory, *n* (%)	281 (46.1)	257 (44.2)	0.52
Major depression, *n* (%)	25 (4.1)	29 (5.0)	0.49
Minor depression, *n* (%)	85 (14.0)	84 (14.5	0.87
Physical activity**, *n* (%)	548 (90.0)	500 (86.1)	0.04
Grip strength (kg), mean (*SD*)	29.2 (9.9)	22.1 (11.7)	<0.0001
Regular gait speed (m/s), mean (*SD*)	1.18 (0.19)	1.08 (0.19)	<0.0001
Max gait speed (m/s), mean (*SD*)	1.64 (0.33)	1.48 (0.31)	<0.0001
Chair-stand (s) mean (*SD*)	13.3 (4.7)	14.1(5.3)	0.008
Balance (s), mean (*SD*)	19.6 (10.9)	12.7 (10.8)	<0.0001

*Cardiovascular disease includes as angina pectoris and/or myocardial infarction.

**Physical activity based on the Saltin–Grimby Physical Activity Level Scale (Grimby et al., 2015), (option 2–4). P-values based on Fischer’s exact test or t-test.

**Table 2:** Associations between the *APOE* 𝜀4 allele and change in physical fitness measures between age 75 and 79.

		*APOE* 𝜀4 carrier mean (*SD*)	*APOE* 𝜀4 non-carrier mean (*SD*)	*p*-value
Grip strength (kg)	*n* = 390	-10.3 (10.8)	-7.8 (10.1)	**0.015**
Regular gait speed (m/s)	*n* = 327	-0.17 (0.16)	-0.14 (0.15)	0.18
Max gait speed (m/s)	*n* = 326	-0.25 (0.26)	-0.24 (0.23)	0.53
Chair-stand (s)	*n* = 389	1.3 (7.1)	1.1 (4.5)	0.78
Balance (s)	*n* = 347	-9.5 (10.1)	-8.9 (10.1)	0.60

*P-values based on linear regression analyses adjusted for sex. Significant p-values are shown in bold.*

**Table 3:** Associations between the *APOE* 𝜀4 allele and measures of physical fitness at age 75 and 79.

		75 year olds				79 year olds		
		*APOE* 𝜀4 carrier mean (*SD*)	*APOE* 𝜀4 non-carrier mean (*SD*)	*p*-value		*APOE* 𝜀4 carrier mean (*SD*)	*APOE* 𝜀4 non-carrier mean (*SD*)	*p*-value
Grip strength (kg)	*n* = 607	28.9 (9.7)	29.4 (9.9)	0.53	*n* = 501	19.6 (10.8)	23.0 (11.9)	**0.006**
Regular gait speed (m/s)	*n* = 597	1.16 (0.20)	1.18 (0.19)	0.35	*n* = 392	1.06 (0.21)	1.09 (0.18)	0.20
Max gait speed (m/s)	*n* = 596	1.64 (0.35)	1.64 (0.32)	1.00	*n* = 390	1.47 (0.35)	1.49 (0.29)	0.69
Chair-stand (s)	*n* = 598	13.4 (5.6)	13.2 (4.4)	0.63	*n* = 496	14.4 (5.2)	14.0 (5.3)	0.50
Balance (s)	*n* = 609	19.3 (11.3)	19.8 (10.7)	0.90	*n* = 445	13.1 (10.7)	12.6 (10.8)	0.56


*P-values based on linear regression analyses adjusted for sex. Significant p-values are shown in bold.*

## Discussion

In this study, we report an association between the *APOE* 𝜀4 allele and larger decline in grip strength between age 75 and 79. This association was independent of cognitive function, as measured with MMSE, and remained after exclusion of cases with dementia. A similar result was seen in cross-sectional analyses in the larger sample of all individuals examined at age 79, showing weaker grip strength among *APOE* 𝜀4 carriers compared to non-carriers. No association with grip strength was seen at age 75, and analyses of the influence of age revealed that the effect of *APOE* 𝜀4 was significantly larger at age 79 compared to age 75.

So far, possible associations between the *APOE* gene and measures of physical function in old age have not been comprehensively investigated, and results have been inconsistent. In a study by Batterham et al. (2013), the 𝜀2 allele was found to be related to a smaller decline in grip strength over a 12-year period. However, after excluding participants with low cognitive scores, the finding became non-significant. The study included individuals aged 70 and older at baseline, but the long follow-up time included the ages examined in our study. In contrast to the findings by Batterham et al. (2013), our result remained significant after correction for cognitive function, and exclusion of individuals carrying the “protective” 𝜀2 allele strengthened the association.

Another study reported associations between the 𝜀4 allele and more rapid motor decline in older individuals (mean age 80 years at baseline) over a period of 10 years (Buchman et al., 2009). The composite measure of global motor function used in that study, included both muscle strength (such as grip strength) and motor performance (such as gait speed), and the association of 𝜀4 with motor decline was for the most part explained by an association with change in muscle strength. In addition, the association between the 𝜀4 allele and motor function increased with age. The results of our study are thus in line with the results from this study, although the participants in the study by Buchman et al. (2009) were a selected group of individuals who agreed to post-mortem donation, while our participants are from a general population.

In our study, no associations were found between *APOE* allele status and gait speed or chair-stand. In contrast, Alfred et al. (2014) studied participants from eight UK cohorts of the HALCyon program, and found associations between the *APOE* 𝜀4 allele and decline in both these measures in one of the cohorts (age 64–82). However, in the cohort including individuals aged 77–80 (the Lothian Birth Cohort 1921), which is most comparable with the ages investigated in our study, no associations with physical performance could be seen. Furthermore, in studies only including tests of chair stand time and/or gait speed, associations with *APOE* 𝜀4 status have been reported in individuals older than 65 years (Melzer et al., 2005) and in men aged 70 years and older (Verghese et al., 2013). Investigation of the relation between *APOE* allele status and physical performance has also been performed in an Asian cohort (Vasunilashorn et al., 2013). No associations were reported, but the proportion of participants with the 𝜀4 allele was much lower in this Asian cohort than in populations of Caucasian origin.

All four measures of physical function (grip strength, gait speed, chair-stand, and standing balance) investigated in this study are predictors of all cause mortality in older individuals from the general population (Cooper et al., 2010). However, the relative value of gait speed, chair-stand, and balance is unclear, since these measures are correlated with each other (Cooper et al., 2010). Grip strength and gait speed are both key components of sarcopenia (Sayer et al., 2013) and frailty (Fried et al., 2001), where grip strength is an estimate of overall muscle strength (Rantanen et al., 1994), while gait speed includes both muscle strength, balance, motor control, and cardiorespiratory function (Studenski et al., 2011). Since grip strength is based on decline in muscle strength only, one might expect to find the clearest association between the *APOE* 𝜀4 allele and this measure, like the result in our study. Still, we cannot give any stable conclusions regarding the possible association between the *APOE* 𝜀4 allele and gait speed, as this test did not include individuals who had home visits at age 79, and persons with the largest decline in gait speed might have been missed.

The mechanism behind the relation between the 𝜀4 allele and motor decline is not clear, but associations have been found with several different diseases and pathologies which can damage the widely distributed motor systems in the brain. One is subclinical vascular disease, e.g., ischemic white matter changes resulting in loss of mobility (Rosano et al., 2006), although cerebrovascular changes may affect gait and balance more than muscle strength. The relation between the 𝜀4 allele and motor decline may also be explained by a common association with AD pathology (Buchman et al., 2009), which has been found not only in brain regions of importance for cognition, but also in basal ganglia related to motor function (Wolf et al., 1999; Burns et al., 2005).

The results of our study do not support any of these hypotheses, since corrections for cardiovascular disease, stroke, BMI, and cognitive function (MMSE) did not influence the association between 𝜀4 status and decline in grip strength over time. In addition, our results did not change after excluding individuals with dementia. A similar result was shown in the study by Buchman et al. (2009), when correcting for vascular factors and mild cognitive impairment. The motor signs that accompany AD often precede a clinical diagnosis (Boyle et al., 2009), and we cannot rule out the possibility that the associations are due to preclinical dementia. However, the results remained also after controlling for cognitive function in individuals without dementia, suggesting that the results were not merely due to incipient dementia. An effect of the *APOE* gene that is independent of cognitive function has also been indicated in a study by Albert et al. (1995), who found an association between the 𝜀4 allele and poorer functional status among non-demented older individuals. However, another study detected an association with functional decline in women only (Blazer et al., 2001), while a third study found a surprisingly lower chance of having ADL disability among women homozygous for the 𝜀4 allele (Kulminski et al., 2008).

Furthermore, previous studies have indicated that a positive effect of physical activity on age-related changes of the brain, such as hippocampal atrophy and amyloid burden, as well as on cognitive decline, is strongest in carriers of the *APOE* 𝜀4 allele (Head et al., 2012; Woodard et al., 2012; Smith et al., 2014). However, when performing the analyses in our study after excluding physically inactive individuals, no main changes of the results could be seen.

The strengths of our study are the representative population-based cohort, the comprehensive examinations performed by trained psychiatric nurses and physiotherapists, and dementia diagnoses made by geriatric psychiatrists. However, there are also limitations. One is the relatively short follow-up time, which makes future dementia impossible to control for. Another is the relatively small sample size, although previous studies of associations between physical function in old individuals and the *APOE* gene are of similar magnitude. Also, some of the individuals were investigated either at the age of 75 or the age of 79, meaning that the cross-sectional analyses partly comprise different persons. Moreover, the examinations of grip strength, chair-stand, and balance were performed both at the clinic and during home visits at age 79, and this might have captured more diseased or frail individuals compared to age 75 when the examinations were performed only at the clinic. However, the association between grip strength and *APOE* 𝜀4 was still significant after excluding individuals examined at home visits.

## Conclusion

Our findings indicate a relation between the *APOE* 𝜀4 allele and grip strength in older individuals. However, despite that the results did not change after controlling for cognitive function and after excluding cases with dementia, we cannot exclude the possibility that the weaker grip strength seen among carriers of the 𝜀4 allele might represent a prodromal symptom of dementia that will appear later in life.

## Author Contributions

AZ and HH designed the study; IS, KF, LJ, and SÖ took part in the acquisition of subjects and data; AZ analyzed the data and all authors took part in the interpretation of the data; AZ and IS drafted the manuscript and HH, KF, LJ, SÖ, KB, and HZ revised it critically for important intellectual content. All authors approved the final version of the manuscript.

## Conflict of Interest Statement

The authors declare that the research was conducted in the absence of any commercial or financial relationships that could be construed as a potential conflict of interest.
